# Association between *ESR1*, *ESR2*, *HER2*, *UGT1A4*, and *UGT2B7* polymorphisms and breast Cancer in Jordan: a case-control study

**DOI:** 10.1186/s12885-019-6490-7

**Published:** 2019-12-30

**Authors:** Laith N. AL-Eitan, Doaa M. Rababa’h, Mansour A. Alghamdi, Rame H. Khasawneh

**Affiliations:** 1 Department of Applied Biological Sciences, Jordan University of Science and Technology, P.O. Box 3030, Irbid, 22110 Jordan; 2 Department of Biotechnology and Genetic Engineering, Jordan University of Science and Technology, Irbid, 22110 Jordan; 3 College of Medicine, King Khalid University, Abha, Saudi Arabia; 4 Department of Hematopathology, King Hussein Medical Center (KHMC), Jordan Royal Medical Services (RMS), Amman, 11118 Jordan

**Keywords:** Breast cancer, Jordanian, ESR, HER2, UGT1A4, UGT2B7

## Abstract

**Background:**

Breast cancer risk, development, and treatment are influenced by genetic variation in certain genes, namely those involved in cell proliferation, tumor suppression, and drug metabolism. In turn, the relevance of the aforementioned genetic variation to cancer depends on the ethnic group in question, highlighting the need for population-specific association studies. Therefore, the objective of the present study was to investigate the association between certain *ESR1*, *ESR2*, *HER2*, *UGT1A4*, and *UGT2B7* single nucleotide polymorphisms and breast cancer.

**Methods:**

Blood samples were collected from 437 Jordanian-Arab breast cancer patients and healthy volunteers and subject to genotyping using the Sequenom MassARRAY® system (iPLEX GOLD).

**Results:**

Our findings show a significant association between breast cancer and the allelic (*P* = 0.02486879) and genotypic (*P* = 0.04793066) frequencies of the *ESR1* polymorphism rs3798577, a result which was confirmed in different genetic models. No other investigated polymorphism showed a significant association with breast cancer itself in Jordanian Arabs, but the Rare Hz (GG) vs Het (AG) genetic model revealed an association of the disease with the *ESR1* polymorphism rs3798577. However, several associations were found between certain polymorphisms and breast cancer’s prognostic factors.

**Conclusion:**

This study suggests that certain polymorphisms may increase the risk of breast cancer in the Jordanian-Arab population. Future research and clinical translation could incorporate the current results in preventative breast cancer approaches tailored for Jordanian-Arab patients.

## Background

Breast cancer (BC) is a complex disease that arises due to a combination of environmental and genetic factors [1]. Current approaches to understanding BC etiology focus on the identification of molecular markers that could aid in the prediction and prognosis of the disease [2, 3]. Mutations in the *BRCA1* and *BRCA2* genes have been well-established as risk factors for BC development, and they are responsible for approximately 90% of the disease’s genetic component [4, 5]. Moreover, certain genetic polymorphisms have been found to modulate the effects of BC chemotherapy, including the selective estrogen receptor modulator tamoxifen, which is prescribed for several BC types. Consequently, polymorphisms in genes implicated in BC pathogenesis, such as those involved in tamoxifen pharmacogenetics, such as the *UGT1A4* and *UGT2B7* genes, are frequent targets of BC research [6, 7].

Excessive endogenous and exogenous estrogen may cause pathological changes in many cancers cell line [8]. estrogen is a key regulator for mammary gland growth and differentiation it is also important in breast carcinoma development and progression [9]. The estrogen receptor 1 (*ESR1*) and estrogen receptor 2 (*ESR2*) genes encode for estrogen receptors alpha (ER-α) and beta (ER-β), respectively, which are activated by estrogen and interact with one another in a dimeric manner [10]. In terms of function, however, ER-α and ER-β appear to have antagonistic functions in breast tissue: ER-α stimulates cell proliferation while ER-β possesses anti-proliferative and tumor-suppressive activity [10, 11]. Thus, genetic variants in genes that encode estrogen receptors such as *ESR* on chromosome 6, could expose a potential risk for breast cancer. Several studies reported that about 55% of ER-positive metastatic BC patients were screened with *ESR1* mutations [12–15].

The *HER2* gene is a Receptor-type tyrosine kinases (RTK) which is a member of epidermal growth factor receptor (EGFR) family that encodes a 185-kDa transmembrane glycoprotein on chromosome 17 [16]. RTK are polymorphic genes that play important role in the regulation of cellular processes [17]. In addition, *HER2* gene involves in human cancers including ovarian [18], bladder [19], lung [20] and stomach [21] cacinomas. In particular, HER2 overexpressed approximately in 30% of BC cases [16]. It also have been reported that overexpression of *HER2* in BC substantially decrease overall survival rates and the metastatic of BC [22, 23].

Lastly, the UDP glucuronosyltransferase 1A4 (*UGT1A4*) and UDP glucuronosyltransferase 2B7 (*UGT2B7*) genes are involved in the elimination of xenobiotics such as tamoxifen, the latter of which loses its anti-estrogenic effects after being glucuronidated by *UGT1A4* and *UGT2B7* [24].

In fact, *ESR1* polymorphisms have been found to be associated with BC susceptibility, although conflicting findings have been presented on whether such polymorphisms increase or decrease the risk of the disease [11]. Similar inconsistent reports have been found for the association between *ESR2* polymorphisms and BC risk [12, 25]. However, due to the carcinogenic effects of *HER2* amplification or overexpression, polymorphisms in the *HER2* gene have been definitively linked with modulated BC risk [26, 27]. Likewise, polymorphisms in the *UGT1A4* and *UGT2B7* genes that lead to their overexpression could lead to rapid tamoxifen metabolism and lower therapeutic effect [28]. Due to the influence of interethnic genetic variation, it would not be accurate to simply extrapolate previously reported results in one population onto another, especially since cancer-related polymorphisms have been reported to have different roles in BC susceptibility and development in different populations [29]. Consequently, the aim of this study is to investigate the association of certain *ESR1*, *ESR2*, *HER2*, *UGT1A4*, and *UGT2B7* single nucleotide polymorphisms (SNPs) with BC susceptibility in the Jordanian-Arab population.

## Methods

### Study subjects and design

Jordanian-Arab BC patients (*n* = 218) and healthy volunteers with patient-matched characteristics (*n* = 219) were enlisted from the Jordanian Royal Medical Services (JRMS) hospital. Participation in the current study entailed the withdrawal of 5 ml of blood from each subject as well as the collection of clinical, demographic, and pathologic data from patient medical records. Written informed consent was obtained from all study subjects, and ethical approval to carry out this study was obtained from Jordan University of Science and Technology’s Institutional Review Board (IRB) with an ethical approval number 14/78/2014.

### DNA extraction and genotyping

Genomic DNA was extracted from each blood sample using the Wizard® Genomic DNA Purification Kit (Promega Corporation, USA) according to the manufacturer’s instructions. The quality and quantity of the purified DNA was ascertained via agarose gel electrophoresis and the Nano-Drop ND-1000 UV-Vis Spectrophotometer (BioDrop, UK), respectively. DNA samples were then diluted with nuclease-free water in order to achieve a final concentration of 20 ng/μl and a final volume ranging between 50 and 500 μl. Afterwards, samples were shipped on ice to Melbourne node of the Australian Genome Research Facility (AGRF) for custom genotyping on the Sequenom MassARRAY® system (iPLEX GOLD) (Sequenom, USA).

### Data analysis

Both the Hardy-Weinberg equilibrium (*p*^*2*^ *+ 2pq + q*^*2*^ *= 1*) (http://www.oege.org/software/hwe-mr-calc.html) and the χ^2^ test were employed to assess the genotypic and allelic frequencies [30]. The genetic association, different genetic models and phenotype-genotype analyses were conducted using the Statistical Package for the Social Sciences (SPSS), version 25.0 (SPSS, Inc., Chicago, IL). For the present study, statistical significance was set at *p-value* < 0.05.

### Correction for multiple testing

According to Li and Ji (2005) a method was used to estimate the effective number of SNPs (*N*_*em*_) that employs a modification of an earlier approach by Nyholt (2004) [31, 32]. Modified Bonferroni procedure was applied to determine a target alpha level (0.05/ *N*_*em*_) that would maintain an overall significance level of 0.05 or less.

## Results

### Candidate SNPs and their minor allelic frequencies

Table 1 lists the *ESR1*, *ESR2*, *HER2*, *UGT1A4*, and *UGT2B7* SNPs investigated by the current study, in addition to the minor alleles of the variants and their frequencies. Genetic variants were selected based on their clinical and pathological significant in addition they were chosen from published polymorphisms associated with BC.

**Table 1:** Minor allele frequencies of gene polymorphisms in breast cancer patients and healthy controls

Gene	SNP ID	Cases (*n* = 218)			Controls (*n* = 219)		
		MA_a_	MAF_b_	HWE_c_ *p*-value	MA_a_	MAF_b_	HWE_c_ *p*-value
*ESR1*	rs3020410	A	0.1	0.44	A	0.08	0.63
	rs3798577	C	0.41	0.33	C	0.48	0.68
	rs2234693	T	0.49	0.34	T	0.49	0.03
	rs9340799	G	0.47	0.5	G	0.46	0.02
*ESR2*	rs1256049	T	0.02	1	T	0.02	1
*HER2*	rs1058808	C	0.32	0.76	C	0.32	0.21
*UGT1A4*	rs12468274	C	0.08	0.37	C	0.07	0.61
	rs2011425	G	0.09	0.23	G	0.09	0.38
	rs6755571	A	0.06	0.54	A	0.05	0.11
*UGT2B7*	rs28365062	G	0.16	0.2	G	0.17	0.47
	rs4348159	T	0.16	0.13	T	0.17	0.13

^a^MA: minor allele. ^b^MAF: minor allele frequency. ^c^HWE: Hardy—Weinberg equilibrium. *N/A* not applicable

### Association between BC and ESR1, ESR2, HER2, UGT1A4, and UGT2B7 SNPs

Table 2 summarizes the findings of the present study with regard to genetic association with BC. A correlation was found between BC and the allelic (*P* = 0.02) and genotypic (*P* = 0.04) frequencies of the *ESR1* polymorphism rs3798577. Regarding this, the distribution of the variant allele of the aforementioned SNP (C) within cases were slightly higher than it among control 48 and 41% respectively. Suggesting that the C allele of ESR1 gene variant ‘rs3798577’ may be considered as BC risk factor.

**Table 2:** Association of the investigated *ESR1*, *ESR2*, *HER2*, *UGT1A4*, and *UGT2B7* SNPs and breast cancer (BC)

Gene	SNP ID	Allelic and Genotypic Frequencies in Cases and Controls				
		Allele/Genotype	Cases (*n* = 218)	Controls (*n* = 219)	P-value	Chi-square
*ESR1*	rs2234693	C	222(0.51)	221(0.51)	0.943	0.005
		T	216(0.49)	213(0.49)		
		CC	60 (27.4)	48 (22.1)	0.069	5.328
		TC	102 (46.6)	125 (57.6)		
		TT	57 (26)	44 (20.3)		
	rs9340799	A	231(0.53)	234(0.54)	0.782	0.076
		G	205(0.47)	200 (0.46)		
		AA	64 (29.4)	54 (24.9)	0.067	5.383
		AG	103 (47.2)	126 (58.1)		
		GG	51 (23.4)	37 (17.1)		
	rs3020410	C	399(0.9)	399(0.92)	0.387	0.748
		A	43(0.1)	35(0.08)		
		CC	181 (81.9)	184 (84.8)	0.698	0.718
		CA	37 (16.7)	31 (14.3)		
		AA	3 (1.4)	2 (0.9)		
	rs3798577	T	258(0.59)	224(0.52)	0.024	5.033
		C	178(0.41)	210(0.48)		
		TT	80 (36.7)	56 (25.8)	0.047	6.076
		TC	98 (45)	112 (51.6)		
		CC	40 (18.4)	49 (22.6)		
*ESR2*	rs1256049	C	434(0.98)	425(0.98)	0.777	0.08
		T	8(0.02)	9(0.02)		
		CC	213 (96.4)	208 (95.8)	0.774	0.082
		CT	8 (3.6)	9 (4.2)		
*HER2*	rs1058808	G	300(0.68)	296(0.68)	N/A	N/A
		C	140(0.32)	138(0.32)		
		GG	101 (45.9)	105 (48.4)	0.503	1.372
		GC	98 (44.5)	86 (39.6)		
		CC	21 (9.6)	26 (12)		
*UGT1A4*	rs12468274	T	400(0.92)	402(0.93)	0.627	0.236
		C	36 (0.08)	32(0.07)		
		TT	182 (83.5)	185 (85.2)	0.611	0.258
		CT	36 (16.5)	32 (14.8)		
	rs2011425	T	399(0.91)	392(0.91)	0.974	0.001
		G	39(0.09)	38 (0.09)		
		TT	180 (82.2)	177 (82.3)	0.974	0.001
		TG	39 (17.8)	38 (17.7)		
	rs6755571	C	416(0.94)	413(0.95)	0.694	0.154
		A	26(0.06)	23(0.05)		
		CC	196 (88.7)	197 (90.4)	0.638	0.897
		CA	24 (10.9)	19 (8.7)		
		AA	1 (0.4)	2 (0.9)		
*UGT2B7*	rs28365062	A	371(0.84)	362(0.83)	0.605	0.267
		G	69 (0.16)	74(0.17)		
		AA	159 (72.3)	152 (69.7)	0.829	0.374
		GA	53 (24.1)	58 (26.6)		
		GG	8 (3.6)	8 (3.7)		
	rs4348159	C	369(0.84)	361(0.83)	0.785	0.074
		T	71(0.16)	73(0.17)		
		CC	158 (71.8%)	152 (70)	0.860	0.3
		TC	53 (24.1%)	57 (26.3)		
		TT	9 (4.1%)	8 (3.7)		

*P*-Value < 0.05 was considered as significant

**Fig. 1:**
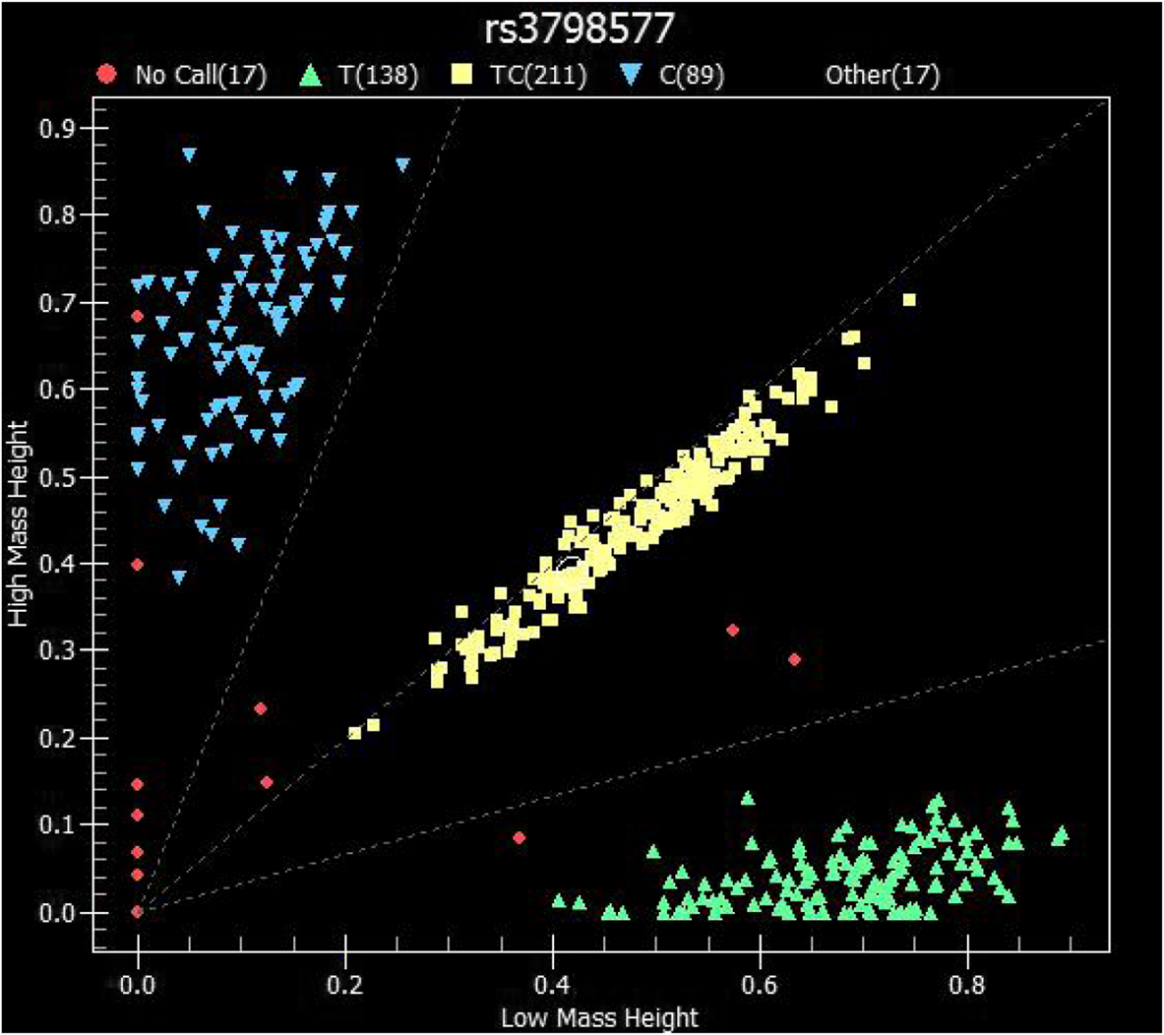
Scatter plot representing Sequenom data for the rs3798577 SNP of the *ESR1* gene. Each dot refers to a single sample, and each color indicates a different genotype

Fig. 1 illustrates the scatter pattern of genotypic distribution for the rs3798577 polymorphism. However, the other investigated *ESR1* and *ESR2* SNPs did not show any significant relationship with BC. Incorporating different genetic models into the association analysis revealed a significant association between BC and the *ESR1* polymorphism rs9340799 for the Rare Hz (GG) vs Het (AG) genetic model (χ^2^ = 4.29). Moreover, a correlation was found between BC and the ESR1 polymorphism rs3798577 for both the Het (CT) vs Common Hz (TT) (χ^2^ = 4.88) and the Rare Hz (CC) vs Common Hz (TT) (χ^2^ = 4.16) genetic models (Table 3). On the other hand, no significant association was found between the investigated *HER2*, *UGT1A4,* and *UGT2B7* polymorphisms and BC in the Jordanian-Arab population sample (Tables 2 and 3).

**Table 3:** Genetic association analysis for the ESR1, HER2, UGT1A4, and UGT2B7 SNPs using different genetic models

Gene	SNP ID	Category Test	Odds Ratio	95% CI	Chi square*
*ESR1*	rs2234693	Het (GT) vs Common Hz (GG)	0.65	0.41–1.04	3.31
		Rare Hz (TT) vs Het (GT)	1.59	0.99–2.55	3.7
		Rare Hz (TT) vs Common Hz (GG)	1.04	0.6–1.79	0.02
	rs9340799	Het (AG) vs Common Hz (AA)	0.69	0.44–1.08	2.67
		Rare Hz (GG) vs Het (AG)	1.69	1.03–2.77	4.29
		Rare Hz (GG) vs Common Hz (AA)	1.16	0.67–2.03	0.28
	rs3020410	Het (CT) vs Common Hz (CC)	1.21	0.72–2.04	0.53
		Rare Hz (TT) vs Het (AG)	1.26	0.2–8.01	0.06
		Rare Hz (TT) vs Common Hz (CC)	1.52	0.25–9.23	0.21
	rs3798577	Het (GT) vs Common Hz (GG)	0.61	0.4–0.95	4.88
		Rare Hz (TT) vs Het (GT)	0.93	0.57–1.53	0.07
		Rare Hz (TT) vs Common Hz (GG)	0.57	0.33–0.98	4.16
*HER2*	rs1058808	Het (GA) vs Common Hz (GG)	1.18	0.8–1.76	0.7
		Rare Hz (AA) vs Het (GA)	0.71	0.37–1.35	1.1
		Rare Hz (AA) vs Common Hz (GG)	0.84	0.44–1.59	0.29
*UGT1A4*	rs6755571	Het (GA) vs Common Hz (AA)	1.27	0.67–2.39	0.55
		Rare Hz (GG) vs Het (GA)	0.4	0.03–4.7	0.57
		Rare Hz (GG) vs Common Hz (AA)	0.5	0.05–5.59	0.33
*UGT2B7*	rs28365062	Het (CT) vs Common Hz (CC)	0.87	0.57–1.35	0.37
		Rare Hz (TT) vs Het (CT)	1.09	0.38–3.12	0.03
		Rare Hz (TT) vs Common Hz (CC)	0.96	0.35–2.61	0.01
	rs4348159	Het (CT) vs Common Hz (CC)	0.89	0.58–1.38	0.25
		Rare Hz (TT) vs Het (CT)	1.21	0.43–3.37	0.13
		Rare Hz (TT) vs Common Hz (CC)	1.08	0.41–2.88	0.03

* For significant association χ2 should be > 3.84 with *P* < 0.025

*CI* indicates confidence interval

### Association of the Clinical and Pathological Factors of BC with ESR1, ESR2, HER2, UGT1A4, and UGT2B7 SNPs

In the present study, a group of known clinical and pathological BC factors were investigated for their association with the *ESR1* and *ESR2* SNPs (Table 4). The *ESR1* SNPs rs3798577 (CC vs CT vs TT) and rs9340799 (AA vs AG vs GG) were associated with family history of BC (*P* = 0.032) and body mass index (*P* = 0.007), respectively. While the *ESR1* SNP rs3020410 (CC vs CA vs AA) was correlated with both estrogen receptor status (*P* = 0.012) and tumor size (*P* = 0.032). The *ESR2* polymorphism rs1256049 (CC vs CT) exhibited an association with age at BC diagnosis (*P* = 0.019).

**Table 4:** Association between different *ESR1* and *ESR2* SNP genotypes and the Clinico-pathological attributes of breast cancer (BC)

Clinical attributes of BC	*ESR1*				*ESR2*
	rs3020410 CC vs CA vs AA	rs3798577 CC vs CT vs TT	rs2234693 CC vs CT vs TT	rs9340799 AA vs AG vs GG	rs1256049 CC vs CT
Age at BC diagnosis ^b^	0.632	0.528	0.179	0.190	**0.019**
Age at first pregnancy ^b^	0.904	0.295	0.128	0.318	0.634
Age at menarche ^b^	0.741	0.866	0.154	0.138	0.570
Age at menopause ^b^	0.965	0.077	0.627	0.664	0.533
Allergy ^a^	0.300	0.893	0.886	0.749	0.625
Body mass index ^b^	0.627	0.209	0.126	**0.007**	0.983
Breastfeeding status ^a^	0.206	0.497	0.895	0.540	0.448
Co-morbidity ^a^	0.914	0.719	0.485	0.615	0.868
Family history ^a^	0.450	**0.032**	0.674	0.706	0.497
Smoking ^a^	0.067	0.722	0.868	0.575	0.415
Pathological attributes of breast cancer (BC)					
Axillary lymph nodes ^a^	0.434	0.314	0.078	0.266	0.805
Estrogen receptor status ^a^	**0.012**	0.398	0.803	0.517	0.569
HER2 ^a^	0.561	0.642	0.152	0.420	0.492
Histology classification ^a^	0.702	0.610	0.818	0.898	0.806
Lymph node involvement ^a^	0.772	0.362	0.318	0.255	0.534
Progesterone receptor status ^a^	0.966	0.756	0.536	0.495	0.736
Tumor differentiation ^a^	0.970	0.399	0.596	0.849	0.056
Tumor size ^b^	**0.032**	0.177	0.637	0.619	0.536
Tumor stage ^a^	0.793	0.158	0.199	0.155	0.614

^a^Pearson’s chi-squared test was used to determine genotype-phenotype association

^b^Analysis of variance (ANOVA) was used to determine genotype-phenotype association

The association between the *HER2, UGT1A4,* and *UGT2B7* SNPs and the clinical and pathological BC factors was also examined (Table 5). The *HER2* rs1058808 (GG vs GC vs CC) SNP was associated with both progesterone receptor status (*P* = 0.01) and tumor size (*P* = 0.013). Regarding *UGT1A4*, its rs12468274 (TT vs CT) and rs2011425 SNPs were correlated with allergy (*P* = 0.001) and tumor size (*P* = 0.002). However, no such significant association was found between the investigated *UGT2B7* SNPs and the clinical or pathological features of BC.

**Table 5:** Association between different *HER2, UGT1A4,* and *UGT2B7* SNP genotypes and the Clinico-pathological attributes of breast cancer (BC)

Clinical attributes of BC	*HER2*	*UGT1A4*			*UGT2B7*	
	rs1058808 GG vs GC vs CC	rs12468274 TT vs CT	rs2011425 TT vs TG	rs6755571 CC vs CA vs AA	rs28365062 AA vs AG vs GG	rs4348159 CC vs CT vs TT
Age at BC diagnosis ^b^	0.457	0.443	0.677	0.958	0.249	0.242
Age at first pregnancy ^b^	0.712	0.363	0.280	0.593	0.416	0.258
Age at menarche ^b^	0.352	0.733	0.632	0.610	0.303	0.301
Age at menopause ^b^	0.369	0.198	0.257	0.802	0.817	0.477
Allergy ^a^	0.393	**0.001**	0.901	0.820	0.296	0.363
Body mass index ^b^	0.373	0.264	0.177	0.729	0.806	0.796
Breastfeeding status ^a^	0.107	0.424	0.556	0.058	0.839	0.726
Co-morbidity ^a^	0.137^a^	0.2802	0.884	0.936	0.895	0.889
Family history ^a^	0.46	0.882	0.337	0.221	0.418	0.686
Smoking ^a^	0.275	0.380	0.150	0.273	0.667	0.403
Pathological attributes of BC						
Axillary lymph nodes^a^	0.645	0.994	0.607	0.447	0.967	0.451
Estrogen receptor^a^	0.051	0.555	0.583	0.705	0.798	0.121
HER2^a^	0.054	0.223	0.295	0.968	0.223	0.567
Histology classification ^a^	0.786	0.916	0.201	0.535	0. 820	0.927
IHC profile^a^	0.252	0.472	0.409	0.918	0.472	0.826
Lymph node involvement ^a^	0.875	0.368	0.658	0.386	0.769	0.317
Progesterone receptor ^a^	**0.010**	0. 770	0.109	0.422	0.919	0.496
Tumor differentiation^a^	0.288	0.426	0.690	0.373	0.373	0.855
Tumor size ^b^	**0.013**	0.323	**0.002**	0.232	0.359	0.941
Tumor stage ^a^	0.580	0.712	0.347	0.322	0.675	0.788

^a^Pearson’s chi-squared test was used to determine genotype-phenotype association

^b^Analysis of variance (ANOVA) test was used to determine genotype-phenotype association

### Haplotype analysis

The *ESR1*, *ESR2*, and *UGT1A4* SNPs were subject to haplotype analysis. Our results revealed two separate blocks: *ESR* (rs3020410, rs3798577, rs1256049, rs2234693, and rs9340799) and *UGT1A4* (rs12468274, rs2011425, and rs6755571)*.* Table 6 shows the frequency ratios for cases and controls as well as the *p-values* for each block, and no association was deduced between the aforementioned haplotypes and BC risk in the present study.

**Table 6:** Haplotypic analysis of *ESR1, ESR2,* and *UGT1A4* polymorphisms

Haplotype	Frequency of block	Frequency ratio (case:control) (%)	Odds ratio (95% CI)	*P*-value
*ESR1* and *ESR2* Block (rs3020410, rs3798577, rs1256049, rs2234693, and rs9340799)				
CTCCG	0.2417	0.2761: 0.232	1:00	N.A
CTCTA	0.2358	0.2172: 0.2345	0.90 (0.56–1.45)	0.66
CCCCG	0.1957	0.2025: 0.1681	0.61 (0.35–1.04)	0.071
CCCTA	0.1702	0.2008: 0.1538	0.65 (0.42–1.02)	0.061
ACCTA	0.0355	0.0266: 0.0391	0.73 (0.27–1.94)	0.53
ATCTA	0.0355	0.0414: 0.0382	1.16 (0.46–2.89)	0.76
CCCCA	0.0277	0.0291: 0.0274	0.80 (0.31–2.04)	0.64
CTCCA	0.0186	0.0168: 0.0194	0.81 (0.22–2.92)	0.74
Global haplotype association p-value: 0.47				
*UGT1A4* Block (rs12468274, rs2011425, and rs6755571**)**				
TTC	0.8552	0.8557:0.8548	1.00	N.A
CGC	0.0771	0.0726: 0.0816	1.12 (0.65–1.92)	0.69
TTA	0.0526	0.0501:0.0551	1.07 (0.59–1.95)	0.82
TGC	0.0118	0.019: 0.0048	0.25 (0.05–1.21)	0.086
Global haplotype association *p*-value: 0.39				

## Discussion

Studies focusing on breast cancer (BC) genetics are increasingly shedding light on the etiology, progression, and treatment of the disease [33, 34]. However, the presence of genetic differences at the ethnic level mandates that cancer-related polymorphisms reported in one group be similarly investigated for any such association in other groups [35, 36]. This rings true for Arab populations especially, which are neither homogenous in their cancer distribution nor identical in their cancer genetic profiles [37]. Therefore, the aim of the present study was to investigate the association of specific *ESR1*, *ESR2*, *HER2*, *UGT1A4*, and *UGT2B7* SNPs with BC in Jordanian-Arabs.

Our findings show that the *ESR1* polymorphism rs3798577 was significantly associated with BC and history of BC in the Jordanian-Arab population, and it was similarly found to confer higher BC risk in the Tunisian-Arab population [38]. rs3978577 polymorphism is located in the 3′ UTR of ER-α, and it has been suggested to increased the overall risk of BC [25]. Moreover, it has been revealed that T allele of ESR1 rs3798577 serve as binding site for forkhead box transcription factor (FOXP1). FOXP1 is involved in proliferation, differentiation in addition to malignant transformation. Fox et al. (2004) indicated that FOXP1 might act as coregulator of *ESR1* Expression [39]. While C allele may serve as Sex determining region Y-box 5 (SOX5) binding site which is a transcription factor that binds to *ESR1* promoter and play role in embryonic development and determination of the cell fate [40].

In contrast, Ghali et al. (2018) found that the *ESR1* rs2234693 and the *ESR2* rs1256049 SNPs were positively and negatively associated with BC in Tunisian Arabs, respectively, while our results only showed an association between rs1256049 and age at BC diagnosis in Jordanian Arabs [38]. In contrast with our results, the *ESR1* rs2234693 SNP was significantly associated with BC in a meta-analysis covering 44 case-control studies, and different levels of association between the *ESR2* rs1256049 SNP and BC were reported in non-Arab populations [10, 11, 41]. Lastly, no significant association with BC was found for the *ESR1* SNPs rs3020410 and rs9340799 in Jordanian Arabs. However, our results show an association between these SNPs and certain BC prognostic factors: rs9340799 was associated with body mass index while rs3020410 was linked to both estrogen receptor status and tumour size in Jordanian Arabs. In older Caucasian females, the rs9340799 SNP protected against BC, while the C allele of the rs3020410 SNP was associated with increased relapse risk [42, 43].

With regard to the *HER2* gene, it has been well-documented that its overexpression or its amplification can negatively affect BC survival, chemotherapy, and remission [44]. In the present study, no significant association was found between the *HER2* rs1058808 SNP and BC in Jordanian Arabs, but it was significantly associated with progesterone receptor status and tumor size. Conflictingly, this SNP was significantly associated with *HER2* protein expression in Han Chinese BC patients, while another study found no BC association of rs1058808 in the same ethnic group [26, 45]. Moreover, no significant BC association was found for rs1058808 in Mexican and Vietnamese BC patients [46].

In terms of BC pharmacogenetics, the *UGT* genes play an important role in the metabolism of tamoxifen, a first-therapy for several types of BC [24]. Concerning *UGT1A4* and *UGT2B7*, our results showed no significant association between the investigated SNPs and BC in Jordanian Arabs. However, the *UGT1A4* rs12468274 and rs2011425 SNPs were found to be associated with allergy and tumor size, respectively. In Spanish Caucasians, the homozygous mutant form of the rs2011425 SNP was associated with lower concentrations of active tamoxifen metabolites [24].

## Conclusions

In conclusion, it can be seen that the influence of certain *ESR1*, *ESR2*, *HER2*, *UGT1A4*, and *UGT2B7* SNPs on BC in Jordanian Arabs differs from that in other populations. The findings of the present study identified the *ESR1* SNP rs3798577 as being significantly associated with BC, which could potentially be taken into consideration in preventative approaches to BC in the Jordanian population. Further characterization of the role of such variants in specific populations will definitely aid in understanding BC etiology, progression, and treatment.

## Data Availability

The datasets generated and/or analysed over the course of the study are not publicly available but are available from the corresponding author on reasonable request.

## Abbreviations

AGRF: Australian genome research facility
BC: Breast cancer
χ^2^: Chi squared value
DNA: Deoxyribonucleic acid
ESR: Etrogen receptor
HER2: Human epidermal growth factor receptor 2 marker
Het: Heterozygote
HWE: Hardy-Weinberg equilibrium
Hz: Homozygote (Hz)
IRB: Institutional review board
JRMS: Jordanian Royal medical services
PR: Progesterone receptor
SNPs: Single nucleotide polymorphisms
SPSS: Statistical package for the social sciences (SPSS)
UGTs: UDP glucuronosyltransferases
